# Telehealth for Noncritical Patients With Chronic Diseases During the COVID-19 Pandemic

**DOI:** 10.2196/19493

**Published:** 2020-08-07

**Authors:** Na Liu, Robin Huang, Tanya Baldacchino, Archana Sud, Kamal Sud, Mohamed Khadra, Jinman Kim

**Affiliations:** 1 The University of Sydney Business School Sydney Australia; 2 School of Computer Science, The University of Sydney NSW Australia; 3 Telehealth and Technology Centre, Nepean Blue Mountains Local Health District NSW Australia; 4 Department of Infectious Diseases, Nepean Hospital NSW Australia; 5 Department of Medicine (Infectious Diseases and Immunology), Nepean Clinical School, University of Sydney NSW Australia; 6 Nepean Clinical School, Faculty of Medicine and Health, The University of Sydney NSW Australia; 7 Department of Renal Medicine, Nepean Hospital Kingswood, NSW Australia; 8 Strategy and Innovation, Nepean Blue Mountains Local Health District NSW Australia

**Keywords:** telehealth, chronic diseases, COVID-19, coronavirus, pandemic, remote monitoring, copresence

## Abstract

During the recent coronavirus disease (COVID-19) pandemic, telehealth has received greater attention due to its role in reducing hospital visits from patients with COVID-19 or other conditions, while supporting home isolation in patients with mild symptoms. The needs of patients with chronic diseases tend to be overlooked during the pandemic. With reduced opportunities for routine clinic visits, these patients are adopting various telehealth services such as video consultation and remote monitoring. We advocate for more innovative designs to be considered to enhance patients’ feelings of “copresence”—a sense of connection with another interactant via digital technology—with their health care providers during this time. The copresence-enhanced design has been shown to reduce patients’ anxiety and increase their confidence in managing their chronic disease condition. It has the potential to reduce the patient’s need to reach out to their health care provider during a time when health care resources are being stretched.

## Introduction

Given the current coronavirus disease (COVID-19) outbreak, telehealth is being adopted widely in innovative ways, so that patients can access care while maintaining social distancing without risking exposure to and spread of COVID-19. Telehealth also promotes the safety of essential clinical staff. Additionally, using telehealth can avoid unnecessary hospital visits, thereby providing some relief to the currently overstretched health resources [1]. With modern telehealth services, patients can arrange a video consultation (VC) with their general practitioner (GP) or specialists instead of traveling to the clinics [2]. Remote patient monitoring (RPM) and patient engagements are also major components of telehealth, which allow patients to share their health-related data with their health care workers in real time [3] and be involved in shared decision making with improved health literacy (Muscat et al, unpublished data) [4]. One of core enablers of telehealth is the provision of timely intervention to patients based on their real-time health indicators (eg, from RPM) before their next scheduled formal visit or via VC service.

As health care resources may be limited during the pandemic, the allocation of resources will focus on managing acutely sick patients with COVID-19 rather than non–COVID-19 patients [5]. During the pandemic, patients with chronic conditions, such as obesity, diabetes, cardiovascular disease, and kidney disease, are at a higher risk of developing serious complications due to COVID-19 and dying [6]; there are also concerns that missing usual ongoing care can lead to adverse health outcomes.

Another concern is that patients with chronic conditions may be fearful of accessing their usual health care services in order to minimize their risk of infection and complications that may arise from infection [7]. Consequently, patients with chronic conditions in self-isolation are not attending clinic visits that not only provide health care services but also serve as a source of motivation for maintaining adherence to medications and behavior changes, such as diet and exercise, required to treat the chronic condition. With reduced clinic visits, patients’ communication channels with clinicians and nursing staff are cut off, thereby compromising the gains that were obtained with such interactions before the COVID-19 pandemic.

Telehealth for patients with chronic diseases during the pandemic is increasingly important, and we suggest that the innovative adoption of digital technologies can continue to provide valuable patient-clinician communication, not only for clinical care but also for maintaining adherence and behavior changes in patients. We suggest that care providers consider the inclusion of the “copresence” concept, which can be used to enhance the perception of presence and thus the relationship between patients and clinicians in the telehealth era. Copresence refers to a sense of connection with another interactant [8]. It is the perception by a communicator that another person in a mediated or online environment is real, immediate, or present [9]. When a person has a high copresence with others in a digitally mediated communication setting, he or she tends to feel more satisfied with the medium and is more likely to use digital tools. The feeling of copresence does not have to be built through rich or advanced technologies; it can be achieved by building up social cues for the interactions. Copresence was widely studied in the field of human-computer interactions, and its application has been used in the context of online shopping experience [10] and virtual team collaborations [11]. Copresence, when incorporated, can provide patients with the feeling that the clinicians are virtually with them, while not operationally increasing the workload of the clinical staff. We provide three copresence strategies that can be readily applied to ease the burden on care delivery and challenges to patients with chronic diseases during the pandemic.

## Use of Telehealth for Patients With Chronic Diseases During the Pandemic

During the pandemic, telehealth can be used to provide more than conventional VC and RPM by building an efficient copresence-enhanced approach to reduce patients’ anxiety while their face-to-face meeting with GPs are reduced. Additionally, it could help relieve stress among health care workers by reducing the need to constantly monitor patients’ health-related data. During the pandemic, where health care resources are centered around the diagnosis and treatment of COVID-19 patients, it leaves patients with chronic diseases with a potentially longer feedback loop due to the lack of remote clinical support.

### A Copresence-Enhanced Approach

In our previous work, we introduced a concise design to enhance patients’ perception of copresence through the sharing of information and emotions with their clinicians during remote monitoring [12]. The design has been built into a remote monitoring system for patients undergoing hemodialysis at home. The system allows patients to rate their emotions at the end of a dialysis session as part of the self-health reporting exercise, so the clinicians have a general understanding of the patient’s feelings at the end of their dialysis session. Dialysis data of patients expressing a low mood are reviewed as a priority. Patients can also include additional comments for each submission, and clinicians can send feedback (with or without comments) by simply clicking the confirmed function in the system to let patients know their dialysis data, including their self-reported emotions, has been reviewed. The design was shown to reduce patients’ anxiety caused by isolation during remote monitoring. The clinicians and nursing staff also reported satisfaction with the ease sending positive feedback to patients who were doing well with just one click.

During the COVID-19 pandemic, because of the social distancing and isolation rules, this remote monitoring system has become even more useful, as patients on home hemodialysis are able to continue managing their dialysis treatments themselves, without a visit to the dialysis clinic. The data from 65 active patients during past 5 months (February to June 2020) has led to 3166 recorded sessions. There is no increase in negative emotions expressed by patients after their dialysis treatments. In total, 31% of the sessions were recorded with additional comments. Patients continue to report feeling less isolated and lower anxiety levels when they receive feedback from their health care providers via the RPM system.

### Copresence Strategies

#### Alleviating Patients’ Anxiety of Not Being Able to Visit Hospitals

During the pandemic, opportunities for patients with chronic diseases to meet their clinicians are limited despite increased health concerns. With telehealth solutions featuring a copresence-enhanced design, patients can find an easy-to-access channel to share their feelings and thoughts with their health care providers [12]. Allowing patients to express themselves is an important part of clinician-patient communication, and it can be effective in reducing patients’ anxiety and providing comfort [13]. Even without real-time or synchronous communication, patients can still benefit from copresence, and this can result in reduced anxiety caused by disconnection with their health care team. Patients’ confidence is also enhanced when they receive simple, encouraging messages [14]. They can be more mindful about their health and better adhere to recommended regimens and behaviors without constant checks and reminders.

#### Offsetting the Demand for Health Care Teams

The current way of adopting telehealth requires an additional investment in time for health care staff (eg, increased standby time of clinical staff to respond to patients’ needs outside of normal working hours) [15]. Unfortunately, the additional resources needed to monitor patients are not necessarily available, especially during the pandemic. With copresence-enhanced functions, such as a one-click acknowledgment from the health care providers and prioritized response to patients’ records based on their emotions and health-related data, staff can spend less time on nonurgent responses, thus reducing the stress associated with managing patients’ expectations of a real-time connection with them through telehealth [16].

#### Providing Continuality When the Pandemic Ends

Patients with chronic diseases play a central role in the self-management of their medical condition [16] by making changes to their diet and physical activities to cope with their condition over a long period. Telehealth applications for patients with chronic conditions should be built to provide patients with more assistance and support for a positive behavioral change [17]. The copresence-enhanced design might give patients more confidence and assurance through subtle social cues delivered from the telehealth system [12] and has the potential to provide a continuality in nudging the shift from a hospital-centric chronic care model to a patient-centric one when the pandemic ends.

## Conclusions

During the recent COVID-19 pandemic, more attention has been drawn to telehealth to emphasize its role in reducing hospital visits from both COVID-19 and non–COVID-19 patients and supporting home isolation in patients with mild symptoms. The needs of patients with chronic diseases tend to be overlooked during the pandemic. With reduced opportunities for routine clinic visits, patients with chronic diseases are adopting various telehealth services such as VC and RPM. We advocate for more innovative designs to be considered to enhance patients’ feelings of copresence with their health care providers during this time. The copresence-enhanced design has been shown to reduce patients’ anxiety and increase confidence in managing their chronic disease condition. It also has the potential to reduce patients’ needs to engage their health care providers during a time when health care resources are stretched in most countries severely affected by the pandemic.

## Abbreviations

COVID-19: coronavirus disease
GP: general practitioner
RPM: remote patient monitoring
VC: video consultation
